# The Quasi-Biennial Vertical Oscillations at Global GPS Stations: Identification by Ensemble Empirical Mode Decomposition

**DOI:** 10.3390/s151026096

**Published:** 2015-10-14

**Authors:** Yuanjin Pan, Wen-Bin Shen, Hao Ding, Cheinway Hwang, Jin Li, Tengxu Zhang

**Affiliations:** 1 School of Geodesy and Geomatics, Wuhan University, Wuhan 430079, China; E-Mails: pan_yuanjin@163.com (Y.P.); dhaosgg@126.com (H.D.); txzhang521@163.com (T.Z.); 2 State Key Laboratory of Information Engineering in Surveying, Mapping and Remote Sensing, Wuhan University, Wuhan 430079, China; 3 Department of Civil Engineering, National Chiao Tung University, Hsinchu 300, Taiwan; E-Mail: cheinway@mail.nctu.edu.tw; 4 Shanghai Astronomical Observatory, Chinese Academy of Sciences, Shanghai 200030, China; E-Mail: lijin@shao.ac.cn

**Keywords:** GPS time series, ensemble empirical mode decomposition (EEMD), quasi-biennial vertical oscillations, loading effects

## Abstract

Modeling nonlinear vertical components of a GPS time series is critical to separating sources contributing to mass displacements. Improved vertical precision in GPS positioning at stations for velocity fields is key to resolving the mechanism of certain geophysical phenomena. In this paper, we use ensemble empirical mode decomposition (EEMD) to analyze the daily GPS time series at 89 continuous GPS stations, spanning from 2002 to 2013. EEMD decomposes a GPS time series into different intrinsic mode functions (IMFs), which are used to identify different kinds of signals and secular terms. Our study suggests that the GPS records contain not only the well-known signals (such as semi-annual and annual signals) but also the seldom-noted quasi-biennial oscillations (QBS). The quasi-biennial signals are explained by modeled loadings of atmosphere, non-tidal and hydrology that deform the surface around the GPS stations. In addition, the loadings derived from GRACE gravity changes are also consistent with the quasi-biennial deformations derived from the GPS observations. By removing the modeled components, the weighted root-mean-square (WRMS) variation of the GPS time series is reduced by 7.1% to 42.3%, and especially, after removing the seasonal and QBO signals, the average improvement percentages for seasonal and QBO signals are 25.6% and 7.5%, respectively, suggesting that it is significant to consider the QBS signals in the GPS records to improve the observed vertical deformations.

## 1. Introduction

Mass displacements originating from atmosphere, ocean and hydrology within the Earth system give rise to loadings and in turn cause the Earth’s surface deformations. Such deformations are recorded as temporal coordinate variations at global positioning system (GPS) ground stations, which can be used to infer mass displacements and loadings [1,2,3,4,5]. Mass-induced loadings have different spatial and spectral characteristics. For example, the patterns and magnitudes of snow and ice loadings depend partially on latitude, and such loadings often have a distinct annual oscillation [6,7]. Evident seasonal deformations caused by water storage changes are seen by GPS, despite the poorly known distribution of such changes [4]. The non-tidal ocean mass variation also displaces the earth’s surface [8,9], and in general the magnitude of the displacement decreases with the distance of a GPS station to seas.

Among the broad GPS applications, velocities of station coordinates derived from time-dependent and long-time GPS observations have been used to model global plate kinematics and regional tectonics [10,11,12], which require precise separation of seasonal signals from the raw GPS observations to improve the modeling. It has been shown that, a typical GPS time series is dominated by the semiannual and annual oscillations [7,13]. Also, efforts have been made to improve the accuracy of GPS vertical (velocity) components [5,14,15], due to the fact that the vertical component is less precise because of the geometry in GPS positioning and tropospheric delay, but it is significant in studying loading and mass displacement. The time-varying gravity from the mission Gravity Recovery and Climate Experiment (GRACE) has been used for correcting hydrological loading effects in GPS [5,15,16,17]. Nevertheless GRACE has limitations in spatial and temporal resolutions. Also, groundwater extractions will cause local-scaled (at few to tens km) ground deformations and gravity changes that cannot be well seen by GRACE. Therefore, GRACE alone cannot fully correct for the loading effects in GPS.

The primary objective of this paper is to analyze variations in GPS vertical time series by ensemble empirical mode decomposition (EEMD) [18,19,20]. EEMD will decompose a given GPS vertical time series into different intrinsic mode functions (IMFs) that constitute the non-secular variations of the time series. These variations are then removed from the time series to improve the velocity determination by GPS. EEMD is capable of isolating signal components without perfectly sinusoidal form, and can overcome the weakness in the sinusoidal representations of such components [20]. The efficiency of this approach will be assessed based on reduction in weighted root-mean-square (WRMS) variation in time series. We will also model non-tidal loadings caused by changes in atmospheric pressure, ocean bottom pressure and surface hydrology to account for the loading effects at the GPS stations in this paper. A section will be dedicated to demonstrating the hydrology-induced seasonal and quasi-biennial loadings from the time-varying GRACE gravity.

## 2. The Effectiveness of EEMD in Recovering Signal Components with Time-varying Amplitudes

### 2.1. Simulated Time Series with Components of Time-varying Amplitudes

**Figure 1:**
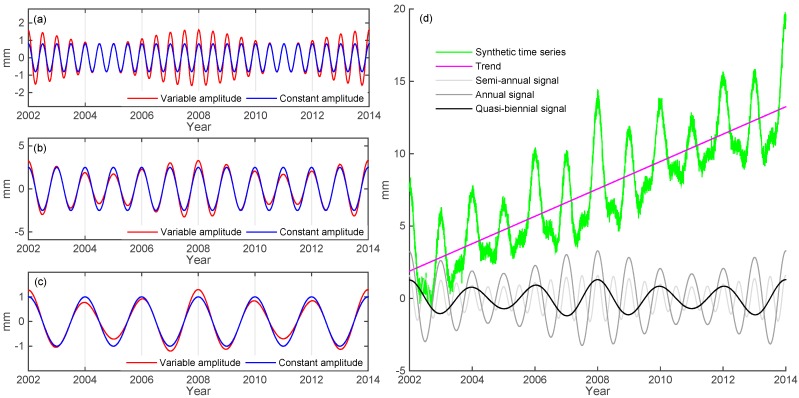
The simulated series (Equation (1), blue), $y_{i}(t)$, with time-invariant (red) and time-varying (red) amplitudes of frequencies of (**a**) 2 cpy, 1.2 mm ± 0.3 mm; (**b**) 1 cpy, 2.5 mm ± 0.3 mm; and (**c**) 0.5 cpy, 1 mm ± 0.3 mm. (**d**) a new synthetic series (green) from the sum of the three synthetic signals from (**a**–**c**), a linear trend and a white noise.

EEMD is an improved method over the method of empirical mode decomposition (EMD) [18,19,20]. Both methods have been widely used in various branches in geoscience to resolve non-stationary and non-linear components in a time series [21,22,23]. Compared to EMD, EEMD reduces the problems of mode-mixing and edge effect [23]. To demonstrate the effectiveness of EEMD, here we carry out a simulation test using a synthetic dataset. First, we generate a time series $y_{i}(t)$ expressed as:

(1) $$y(t)=\sum_{i=1}^{3}A_{i}\cos(2\pi f_{i}t+\varphi_{i})+Bt+v(t)$$

where $A_{i}=a_{i}+b_{i}\sin(tf_{i})$ is instantaneous time-varying amplitudes, $f_{i}$ and $\varphi_{i}$ (i = 1, 2, 3) are given frequencies, B is the coefficient of linear trend, v(t) is data noise. In this paper, we use $f_{1}$ = 2 cycle per year (cpy), $f_{2}$ = 1 cpy, $f_{3}$ = 0.5 cpy, and $\varphi_{1}$ = $\varphi_{2}$ = $\varphi_{3}$ = 0, corresponding to semi-annual (Figure 1a), annual (Figure 1b) and quasi-biennial (Figure 1c) signals, respectively. The time span of the synthetic time series is from January 2002 to December 2012.

Figure 1a–c show the time series of semi-, annual and biennial variations, with time-invariant (blue) and time-varying (red) amplitudes, respectively. Figure 1d shows a series that is the sum of the three time series with time-varying (red) amplitudes, a linear trend and white noise. The given trend is 3 mm/yr and the white noise has a standard deviation of 0.3 cm.

### 2.2. Recovering the Signal Components by EEMD 

In general, a given time series, *f*(*t*), can be decomposed into a number of IMFs, expressed as:

(2) $$f(t)=IMF_{1}(t)+IMF_{2}(t)+IMF_{3}(t)+\cdots +IMF_{n}(t)+r(t)$$

where *n* is the number of IMFs and r(t) is a residual term. Each of the IMFs corresponds to a signal separated from the original observation series. Here we use EEMD to decompose the IMFs corresponding to the signals with time-varying amplitudes as shown by Figure 1 by the following computational procedures [23]:

1. Adding white noise sequence in the target time series;
2. Decomposing the time series containing the white noises into IMFs;
3. Repeating the above two steps, but with different white noise series each time;
4. Taking the mean of many-times decomposed IMFs as the final result IMF.

**Figure 2:**
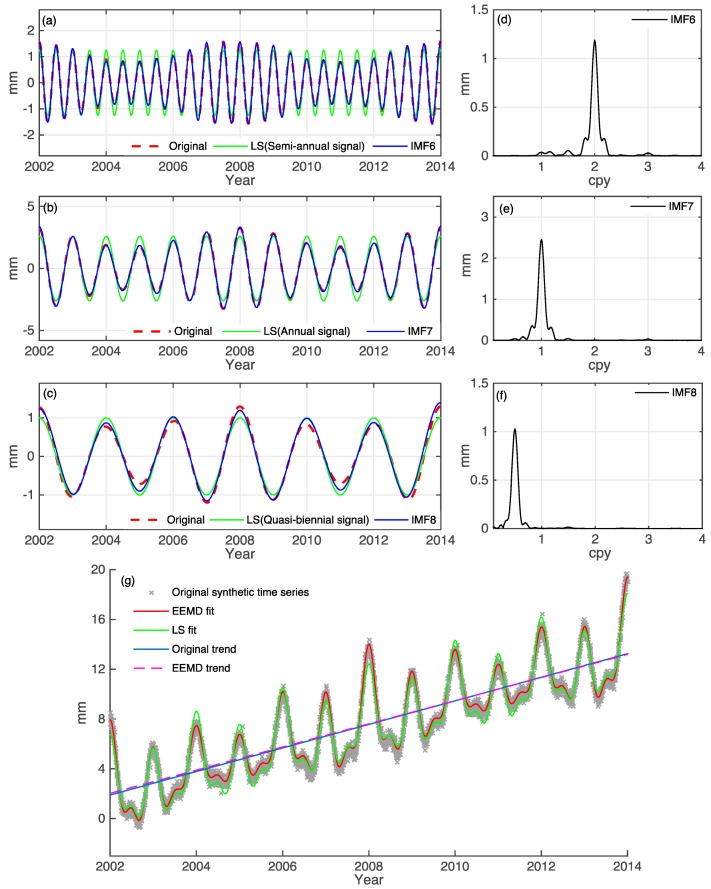
Comparison between synthetic time series (Equation (1)) and the decomposed IMFs. (**a**) the synthetic time series $f_{1}$ (dotted red curve), semi-annual signal by least-squares (LS) (green curve) and IMF6 (blue curve); (**b**) the synthetic time series $f_{2}$ (red dotted curve), annual signal by LS (green curve) and IMF7 (blue curve); (**c**) the synthetic time series $f_{3}$ (red dotted curve), quasi-biennial signal by LS (green curve) and IMF8 (blue curve); (**d**–**f**) are the amplitude spectra of IMF6–8, corresponding to the semi-annual, annual and quasi-biennial signals; (**g**) the original time series with a trend (gray dots), the sum of IMF6, IMF7 and IMF8 with the trend (red curve) and the LS fit (green curve), the original trend (blue curve) and EEMD decomposed trend (dotted purple curve).

Figure 2a–c shows the results from EEMD and least squares (LS), compared to the three known synthetic time series as given by Equation (1) (also see Figure 1). The EEMD decomposes the synthetic time series into 11 IMFs and a trend term, of which three IMFs, *i.e.*, IMF6, IMF7 and IMF8, are identified. It clearly shows that the LS fitting can’t capture well the time variable part, but the IMFs from EEMD do. Figure 2d–f shows the amplitudes of IMF6, 7 and 8, which are consistent with the given amplitudes. Figure 2g shows the sum of IMF6, IMF7 and IMF8 and a linear trend, which matches very well with the original synthetic series. The decomposed trend term filtered by EEMD technique coincide the original one very well, and can be used as an important parameter for velocity, due to the fact that the residual error and nonlinear signals have been removed. Our test here confirms that EEMD has a strong ability to identify the non-linear signal components from a combined times series than the LS method, and the latter is frequently used by various authors. Deficient in using the LS method to identify the signals from a raw time series is that uncertainty about the frequency of unknown signals. However, the EEMD can better reduce noise and extract signals adaptively. Comparison between EEMD and LS fit is provided in Section 4.3. 

## 3. GPS Data Processing Strategy for Optimized Coordinate Solutions

We use the GIPSY/OASIS-II (version 6.2) software developed by the Jet Propulsion Laboratory (JPL, La Canada Flintridge, CA, USA) [24] to process the GPS data. Most of the GPS data are downloaded from the International GNSS Service (IGS) website (available online [25]), and some are downloaded from regional GPS network, including Chinese continental tectonic environment monitoring network. We choose GPS data spanning from 2002 to 2013, and the data process strategy is as follows [15].

To optimize the coordinate solutions, we use the GPS final, non-fiducial daily products of JPL, including satellite orbit and clock estimates, transformation parameters, polar motions, GPS satellite eclipse times, calibration files for IGS ANTEX antennae, available at the JPL archive site (available online [26]). To improve our daily solutions, we corrected for tropospheric delays using the GMF tropospheric mapping function, and *a priori* dry tropospheric delay estimates of the Global Pressure and Temperature (GPT) model [27]. The ocean tide loading was corrected using the FES2004 tide model [28]. FES2004 adopted the amplitudes and phases of the tide-induced diurnal and semi-diurnal deformations predicted by [28], who used Farrell’s elastic Green’s functions [29] for the tidal convolution to obtain surface deformation. The amplitude and phase of the surface deformations at the diurnal, S1, and semi-diurnal, S2, bands are computed as the convolutions of the FES2004 ocean tide and the elastic Green’s functions [28,29]. The surface deformations are computed at an interval 300 s with respect to the instantaneous center of the mass of the whole Earth system (CM). The initial solutions result in the constrained relaxation coordinates of the stations (reference), which are then transformed to the coordinates in the IGS08 frame using the 7-parameters Helmert transformation [30].

For a best resolution of the nonlinear signals, a continuous, outlier-free GPS time series is important. The initial time series contain data gaps and outliers. In this study, the largest initial data gap is 30 days. By testing spline and linear interpolation methods to fill data gaps, we found no significant difference for detecting long-period (e.g., annual or biennial) signals. Hence, we linearly interpolate the gaps using the values from the neighbor values. The outliers were removed using a smooth filter. 

## 4. Results of Nonlinear Signal Identification by EEMD

### 4.1. Global GPS Stations with the Quasi-Biennial Oscillation Signals

**Figure 3:**
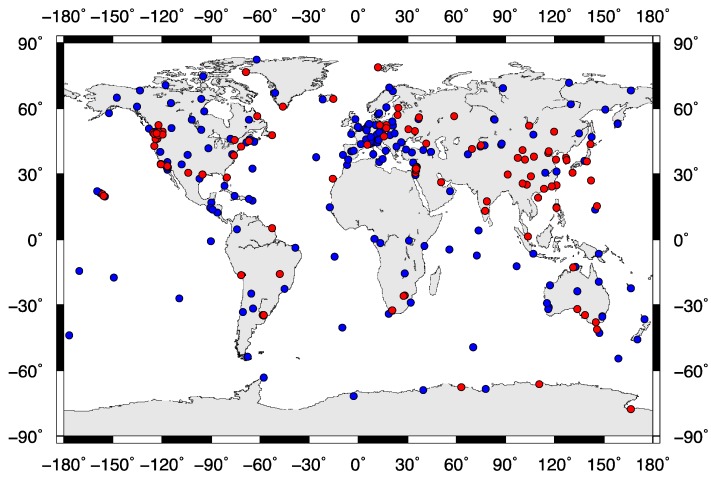
269 GPS stations over the globe are tested from the IGS and regional networks. The red doted 89 sites have both the seasonal and quasi-biennial signals, and the blue doted sites have only the seasonal signals.

After the optimized coordinate solutions in Section 3, we obtained GPS vertical time series at selected IGS core GPS stations and regional GPS continuous stations. We then extracted the nonlinear signals (e.g., seasonal and inter-annual oscillation signals) from the GPS time series using EEMD. As stated in Section 3, the preprocessed GPS time series are continuous and have good quality, so the extracted nonlinear signals are reliable. We analyzed 269 globally distributed GPS stations, including the IGS and regional continuous GPS stations with observations more than 10 years. All of the stations have seasonal signals, of which 89 GPS stations have both the seasonal and quasi-biennial signals (see Figure 3). Note that, most GPS stations in the world see the seasonal variation, and the stations as shown in Figure 3 are characterized by both the seasonal and quasi-biennial signals. The majority of the GPS stations in Figure 3 are located near the seas, with some stations located in southern Alaska and southwestern China. A previous study [7] used GPS and GRACE observations to detect seasonal hydrological loadings in southern Alaska, and demonstrated that such loadings contribute significantly to surface deformations there. In southwestern China, especially the Tibetan Plateau, GRACE has detected mass losses caused by hydrology changes [31], which reduce the resource water for Yangtze River. The key contribution of the seasonal variability for GPS nonlinear oscillation is the regional water resource change, which also possesses pronounced inter-annual variability.

### 4.2. Extracting Nonlinear Signals by EEMD: Example at Lhasa and Kunming 

**Figure 4:**
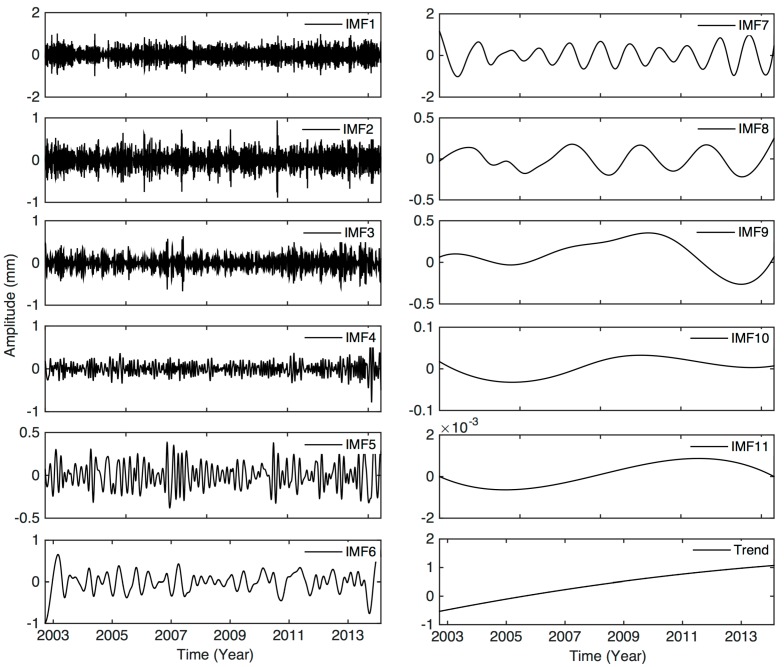
The signals corresponding to IMF1 to IMF11, and a long-term trend, from the GPS vertical time series at station LHAZ, Tibet.

Here we show an example of EEMD to extract the nonlinear signals at station LHAZ, located in Lhasa, Tibet. The GPS time series was decomposed into IMF1 to IMF11 and a secular trend, as shown by Figure 4. The first five IMFs, IMF1 to IMF5, originate from high-frequency signals, and IMF6, IMF7, IMF8 and IMF9 to IMF11 correspond to the semi-annual, annual, quasi-biennial oscillations, and the secular terms, respectively. The time-dependent amplitudes of the IMFs at different frequencies suggest that the nonlinear signals vary with time.

**Figure 5:**
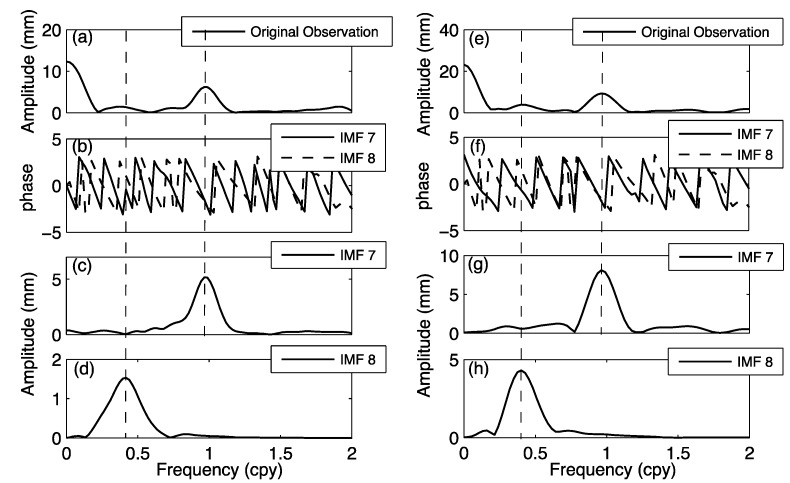
(**a**–**d**) The decomposed signals at LHAZ and (**e**–**h**) at KUNM GPS stations as IMF7 and IMF8 correspondent their phases; (**a**,**e**) are the spectrum of original GPS time series; (**b**,**f**) are the phase of annual and quasi-biennial signals; (**c**,**g**) are amplitude spectrum of annual signal; (**d**,**h**) are amplitude spectrum of quasi-biennial signal.

The high-frequency signals (e.g., IMF1-IMF6) and target signals (e.g., IMF7 and IMF8) with time-dependent amplitudes have been successfully isolated by applying EEMD to original data series. The resulting IMF components have physical meanings explained below. Figure 5 shows the spectra of the annual and quasi-biennial signals (corresponding to IMF7 and IMF8). As expected, the annual signals have the maximum amplitude. Here we focus on the quasi-biennial signals, which was not stated in the GPS community. The signal was not paid attention before because it is weak and hard to identify by the conventional Fourier method. EEMD can extract a weak signal from a complicated series, which is demonstrated for the station at LHAZ as an example.

Another example of EEMD identification of quasi-biennial signal is given at station KUNM, located in Kunming in southwestern China. Both the magnitudes of the quasi-biennial signals at LHAZ and KUNM are about 5 mm, which can be overwhelmed by disturbing signals by hydrology and GPS data noises. Because the quasi-biennial signal will bias the estimation of velocity at a GPS station, we suggest that this signal should be removed before computing the vertical velocity from a given GPS time series. EEMD plays a key role in detecting the quasi-biennial signal. 

We use the autoregressive (AR) method [23,32,33] to estimate the frequency and amplitude values of the target signals (IMF7 and IMF8). The frequencies of the quasi-biennial signals at LHAZ and KUNM are 0.417 ± 0.019 cpy and 0.424 ± 0.019 cpy, respectively, and their amplitudes are 1.530 ± 0.11 mm, and 4.307 ± 0.13 mm. Furthermore, at LHAZ and KUNM, the frequencies of the annual signal are 0.976 ± 0.01 cpy, 0.968 ± 0.006 cpy, respectively, and their amplitudes are 5.169 mm and 8.095 mm.

### 4.3. Comparison between Dominant Signals Recovered by EEMD and Least-Squares Fit

**Figure 6:**
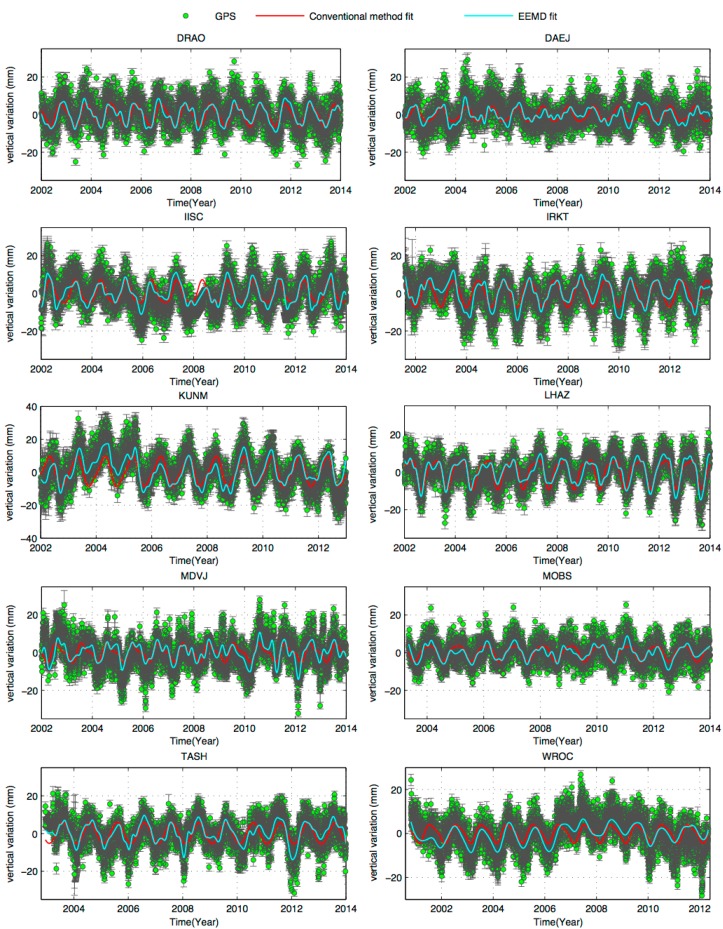
The original vertical GPS time series (green) with one sigma-error bars. The de-trended series formed by IMF6 + IMF7 + IMF8 is given in light-blue, and the red curve is from the LS fitting by the annual and semi-annual sinusoidal terms.

The purpose of identifying the biennial oscillation is for: (1) revealing the nature of this oscillation and (2) for correcting this effect to improve the vertical velocity. A typical method to correct for the seasonal variation in a given GPS vertical time series is LS fitting the series by sinusoidal functions, which is then removed from the original time series. Other methods are sine fitting and linear fitting; examples of such fittings are given in references [17,20]. However, some potential seasonal loadings do not exhibit a perfect sinusoidal form due to the complexity of seasonal signals, therefore the methods mentioned above cannot fit well. An example of LS fitting the series was provided in Section 2.2. To solve this problem, we choose some continuous GPS stations, whose data lengths are longer than 10 years and the magnitudes of seasonal vertical variations exceed 10 mm. By EEMD, the time series were decomposed into IMFs. The IMFs, IMF6, IMF7, and IMF8 and the secular term, IMF9 to IMF12, are combined to form a series. Figure 6 compares this combined series and the series from the LS fit by sinusoidal functions. Figure 6 shows that the combined series from IMFs and the trend is more consistent with the original series than the series from the sinusoidal-function fitting. We see that the series from IMFs capture all the peaks in the original series, but the LS fitted series do not. Table 1 shows the frequencies and amplitudes of the quasi-biennial signals estimated by the AR logarithm.

## 5. Discussion

In the GPS data processing, we did not remove the non-tidal loading effects due to atmospheric pressure, ocean bottom pressure and surface hydrology. Here we model these non-tidal loading effects to explain the decomposed signals, especially the QBO signals, in GPS time series.

### 5.1. Mass Loading Contributions

Nonlinear signals of GPS time series originate from the variations in the surface fluid mass loads whose principal components are tide and non-tidal ocean as well as atmosphere loadings [34]. The accuracy of GPS station coordinates, especially in heights, depends on how well one can effectively remove signals with different periods caused by these surface deformations. To show that the seasonal and quasi-biennial signals indeed exist in the GPS time series, we use global loading models to compute surface deformations. The ocean tide-induced deformations are expressed in the system of the center of the mass of the whole Earth system (CM).

**Table 1:** The frequency and amplitude of quasi-biennial signal at selected GPS stations.

Station	Latitude	Longitude	Frequency (cpy)	Amplitude (mm)
DRAO	−119.625	49.323	0.429 ± 0.015	0.87 ± 0.15
DAEJ	127.374	36.399	0.411 ± 0.013	1.46 ± 0.11
IISC	77.57	13.021	0.419 ± 0.015	1.97 ± 0.15
IRKT	104.316	52.219	0.468 ± 0.009	1.55 ± 0.13
KUNM	102.797	25.030	0.424 ± 0.019	4.30 ± 0.13
LHAZ	91.104	29.657	0.417 ± 0.019	1.53 ± 0.11
MDVJ	37.214	56.021	0.458 ± 0.019	1.63 ± 0.17
MOBS	144.975	−37.829	0.429 ± 0.015	0.52 ± 0.11
TASH	75.23	37.77	0.421 ± 0.01	2.38 ± 0.14
MROC	17.062	51.113	0.439 ± 0.012	1.59 ± 0.13

**Figure 7:**
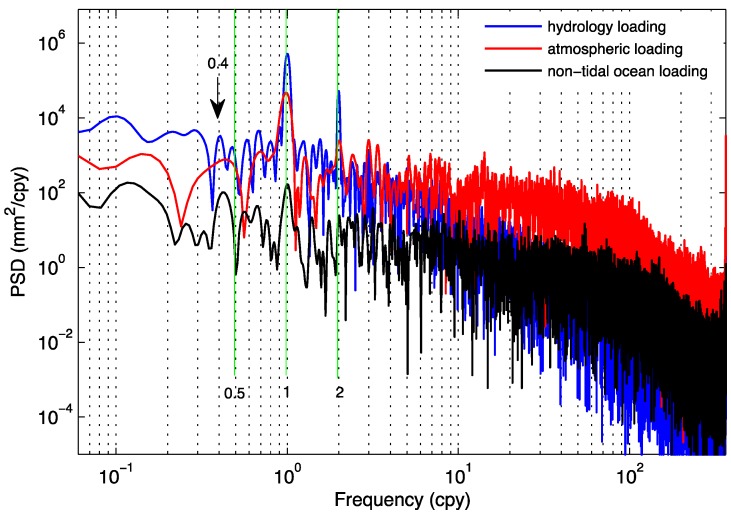
Power spectra density (PSD) of hydrology loading, atmosphere pressure loading and non-tidal ocean loading at LHAZ region corresponding to LHAZ GPS station in China. Blue, red and black curves denote the PSDs of the hydrology loading, atmosphere pressure loading, and non-tidal ocean loading, respectively.

To model the seasonal and the nonlinear signals seen in the GPS time series, we use three global loading models to obtain the loading deformations caused respectively by the atmospheric pressure change, non-tidal oceanic change and hydrologic change. These global loading models are respectively ECMWF operational model (3 h, 0.25 × 0.25°), ECCO1 (kf080) ocean bottom pressure model (12 h, 1 × 1°) and MERRA-land model (1 h, 0.50 × 0.67°) released by GGFC (Global Geophysical Fluid Center), which provide grid-based surface deformation information in time series (available online [35]). The surface deformations caused by the loads are calculated by convolving Farrell Green’s function [29] derived on the basis of the preliminary reference Earth model (PREM) [36]. By applying linearly interpolation and linear interpolation to grid and time period respectively, we obtain the time series of the loading deformations that are coincident with the locations and times of the GPS observations. Figure 7 shows the power spectrum density (PSD) of the three loading time series at station LHAZ (see Figure 6). 

Figure 7 suggests that the PSDs of the three loadings follow the inverse power laws that are typical for a stochastic process, with a significant amount of power near the annual frequency. The PSD of the time series reflects the PSD of the seasonal amplitudes. The PSD of the time series behaves as an inverse power law for high frequencies. Comparison of the blue curves with the red one as shown in Figure 7 demonstrates that a stochastic seasonal process is justified. The PSDs for atmosphere pressure and non-tidal ocean loading show the annual signals obviously. The power order of atmosphere pressure loading is larger than the non-tidal ocean loading, showing respective effects on GPS coordinate time series. The quasi-biennial signal was discovered from the PSD of atmosphere pressure loading, however, it was not found in non-tidal ocean loading.

### 5.2. Comparison with GRACE-Derived Loading 

**Figure 8:**
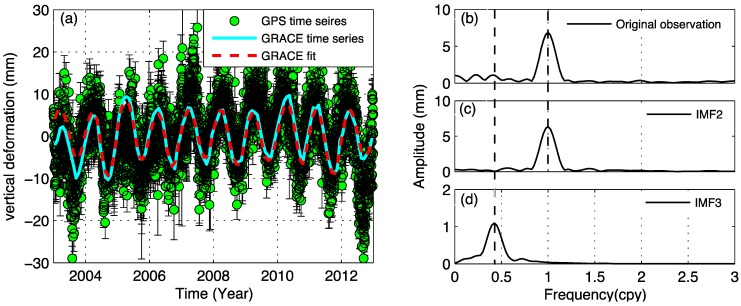
The example de-trended time series for continuous GPS site LHAZ (green error bar), monthly displacement time series of GRACE result is cyan curve in (**a**) correspondent to its amplitude spectrum curve in (**b**–**d**) are annual and quasi-biennial signals decomposed by EEMD. The GRACE fit curve is added by IMF2 and IMF3.

Considering the unreliability of the global loading models for some regional place, we compute the displacement of total hydrology loading from GRACE results to further confirm the quasi-biennial signal. In this study, we use the monthly GRACE-derived Stokes coefficients $C_{lm}$ and $S_{lm}$ (of degree *l* and order *m*). The newly spherical harmonic coefficients (up to degree and order 60) of Level-2 Release 05 products were derived by Center for Space Research (CSR) group, time span from year 2002 to 2013, where the tidal and non-tidal ocean with the atmosphere pressure loading effects have been removed. The C20 term was well constrained by Satellite Laser Ranging (SLR) results [37], and the degree-1 components were replaced by Stokes coefficients [38]. The products were applied to the elastic displacement caused by the changing surface mass load [16]:

(3) $$\text{Δ}h(\theta ,\phi )=R\sum_{l=1}^{\infty }\sum_{m=0}^{l}{\bar{P}}_{lm}(\cos\theta )\cdot [C_{lm}\cos(m\phi )+S_{lm}\sin(m\phi )]\cdot \frac{h_{l}^{'}}{1+k_{l}^{'}}$$

where *R* is the Earth radius, ${\bar{P}}_{lm}$ are fully normalized Legendre functions for degree *l* and order *m*; $C_{lm}$ and $S_{lm}$ are spherical harmonic coefficients of the gravity field, $h_{l}^{'}$ and $k_{l}^{'}$ are adopted load Love numbers provided by Farrell [29], which are computed relative to the center of mass of solid earth [15]. Gaussian smoothing of 300 km is applied without using any decorrelation filtering. The loading deformation time series of hydrology in LHAZ position is compared with LHAZ GPS de-trended time series, as shown in Figure 8. The EEMD decomposed results (annual and quasi-biennial signals) are shown in Figure 8c,d. We see a good seasonal correlation between GPS and GRACE signals. Figure 8a indicates that the main hydrology effects make a great contribution to surface deformation. Figure 8d suggests that GRACE time series contain quasi-biennial signals.

### 5.3. Other Potential Causes of Quasi-Biennial Signals

The interannual variability of GPS time series, especially the QBO, has been examined in stratospheric layer where in some regions it is very intensive [39,40,41]. In addition, ocean-atmosphere climate models show that there exists a universal QBO signal [42]. The connections between the stratospheric QBO and tropospheric circulation over Asia in northern autumn suggests that the QBO may be linked to tropospheric circulation over Asia through wave activities and convective activities in autumn, and the wave activities in the low latitudes associated with the QBO seem to be connected with the rainfall distribution over the Asian monsoon region [43]. 

Recent studies [44,45,46] reveal that a QBO signal is also found in ionospheric parameters and geomagnetic activity indices. QBO signals might have potential influences on the length of day (LOD) and polar motion (PM) [47], and consequently influence the GPS observations. However, GPS data processing is complying with the updated standard IERS models that have considered the tropospheric and ionospheric effects as well as the polar tide effect but without considering some kinds of special signals, such an the QBO signals. Hence, some kinds of residual signals especially the QBO signals still remain in the GPS residual time series, which contaminates the application of GPS time series in determining for instance the plate motion.

### 5.4. Correlation Analysis

GPS time series often contain some linear and nonlinear signals which have a large impact on GPS vertical rate and transformation parameters that are used to establish an international terrestrial reference frame or a regional reference frame. The conventional method to remove these signals is using filtering methods. However, the filtering methods may change the nature of the original signal, introducing artificial effects. To overcome this drawback, we extract these signals using EEMD method, and then subtract them from original GPS vertical time series. It is an approach that not only ensures the integrity of the original time series, but also help us analyze the effect of nonlinear signal on GPS coordinate positioning accuracy. In our study, we compute the weighted root-mean-square (WRMS) reduction rates to evaluate the correlation between GPS time series and nonlinear signals, using the following formula [15]:

(4) $$WRMS_{reduction}=\frac{WRMS_{GPS}-WRMS_{GPS-Signals}}{WRMS_{GPS}}$$

(5) $$WRMS=\sqrt{\frac{\frac{n}{n-1}\sum_{i=1}^{n}\frac{{\left(P_{i}-P\right)}^{2}}{\sigma_{i}^{2}}}{\sum_{i=1}^{n}\frac{1}{\sigma_{i}^{2}}}}$$

where *n* is number of days, $P_{i}$ is the estimate of the component on *i*-th day, P is the weighted average of the component estimate over all days, and $\sigma_{i}$ is the formal error. With Equations (4) and (5) we can evaluate the GPS time series precision.

**Figure 9:**
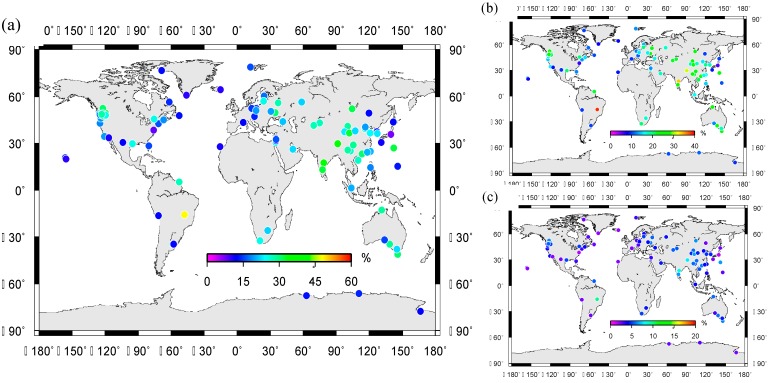
WRMS reduction rates for GPS de-trended vertical time series after removing seasonal and quasi-biennial signals de-trended displacements. The color bar denotes percentage of WRMS reduction rate after signals removed from GPS time series. (**a**) The WRMS reduction rate after removing the seasonal (annual + semi-annual) and quasi-biennial signals; (**b**) The WRMS reduction rate after removing only the seasonal signals; (**c**) The WRMS reduction rate after removing only the quasi-biennial signals.

The nonlinear signals, including annual, semi-annual and quasi-biennial signals are removed from the GPS vertical time series for the correlation analysis. As it can be seen from Figure 9a, the WRMS reduction rates are plotted for continuous GPS stations, which show obvious WRMS improvements. The WRMS of GPS stations are all decreased to a certain extent from the minimum, 7.1%, to the maximum 42.3%, and the average improvement percentage for the selected 96 stations is 33.1%. Figure 9b, the WRMS reduction rate indicates that seasonal signals are the principal components in GPS time series. In Figure 9c we highlight QBO signals in GPS time series. After removing the seasonal and QBO signals, the average improvement percentages for seasonal and QBO signals are 25.6% and 7.5%, respectively. The expected WRMS mainly reflects the basic noise level without residual seasonal effects [15]. Loading effects contribute to the surface elastic deformations, which are included in GPS time series as nonlinear signals. The remaining noise in the corrected time series arises from a combination of longer term correlated noise, errors in the seasonal hydrological corrections, and interannual variations in the load. We note that, besides the seasonal and quasi-biennial signals, other kinds of signals, such as relatively long-period signals also exist in original GPS vertical time series.

## 6. Conclusions

The solid Earth surface deformation information due to redistributions of fluid mass loads is contained in GPS vertical time series. The seasonal variation of GPS time series reflects the seasonal activities of various loading processes, which provides critical information for geophysical sources. In order to separate the target signal from the observation time series, we apply EEMD technique to the GPS time series analysis. By decomposing the GPS time series into different signals contained in different IMFs, we successfully detected the quasi-biennial signals (including their frequencies and amplitudes), which have obvious impact on their vertical variation in some GPS stations. Hence, the quasi-biennial signals should be considered in relevant studies. We provide the PSD for atmosphere pressure and non-tidal ocean loading and also with the hydrology loading effects. The loading effects and GRACE results suggest that the nonlinear variations (e.g., seasonal and quasi-biennial signals) from GPS vertical time series are caused by various kinds of loading effects. In different frequency range, the strength of a signal is subject to the space distribution. Our study suggests that the quasi-biennial signal is closely related to atmosphere pressure loading. However, the mechanism of quasi-biennial signal is still open. 

To model the GPS vertical time series, we add up these signals (semi-annual, annual and quasi-biennial signals) to fit the GPS time series. Compared with the conventional fitting method (e.g., LS fitting method), our approach based on EEMD fits the observations better. The added curves of several decomposed signals conform to the original GPS time series very well. Assessing the accuracy of vertical variation by calculating the WRMS reduction rates of the GPS time series, we find that the WRMSs of the GPS time series recorded from many stations decrease obviously to different extents after the seasonal and quasi-biennial signals are removed. 
